# Acupressure to improve sleep quality of older people in residential aged care: a randomised controlled trial protocol

**DOI:** 10.1186/s13063-020-04286-2

**Published:** 2020-04-25

**Authors:** Nant Thin Thin Hmwe, Graeme Browne, Lyndall Mollart, Viv Allanson, Sally Wai-Chi Chan

**Affiliations:** 1 School of Nursing and Midwifery, The University of Newcastle, University Drive, Callaghan, NSW 2308 Australia; 2 Maroba Caring Communities, Waratah, NSW Australia; 3 UON Singapore, International and Advancement Division, The University of Newcastle, Callaghan, NSW Australia

**Keywords:** Acupressure, Older people, Aged care, Residential aged care facilities, Sleep disturbance, Sleep quality

## Abstract

**Background:**

Sleep disturbance in older people is an important health issue that is associated with many other health problems. Effective interventions are required to address sleep problems in this group. Acupressure has been suggested as a potential therapeutic technique to improve sleep. The aim of this study is to evaluate the effects of an acupressure intervention on sleep quality, anxiety, depression, and quality of life among older persons in residential aged care facilities within an Australian context.

**Methods/design:**

This is a randomised controlled trial with two parallel groups in a pre- and post-test study. Study participants will be older people living in residential aged care facilities. They will be assigned to intervention and control groups using computer-generated random numbers. The intervention group will receive 12-min acupressure three times per week for four consecutive weeks, applied on three acupoints: HT7, PC6, and SP6. The control group will receive only routine care. The primary outcome measure is sleep quality, and secondary outcomes include anxiety, depression, and quality of life. Data will be collected before the intervention, immediately after the end of the intervention, and 2 weeks after the intervention.

**Discussion:**

This study targets a poorly met healthcare need of older people who are experiencing sleep disturbance and its negative consequences. To the researchers’ knowledge, this is the first study evaluating acupressure in an Australian aged care context, using specific acupoints and a standardised acupressure protocol. If the results show positive outcomes, acupressure could be used to manage sleep disturbances for older people in the Australian context as well as in the global ageing population. This will contribute to reducing negative consequences of sleep disturbance and use of sleep medications.

**Trial registration:**

Australian New Zealand Clinical Trials Registry: ACTRN12619000262101. Registered on 20 February 2019.

## Introduction

Sleep disturbances increase with advancing age, with the prevalent rate approaching 50% in older people aged 65 and above [1]. Sleep disturbances in older persons are associated with poor health status, cognitive decline, depression, poor quality of life (QoL) and higher risk of falls [1, 2]. Recent systematic reviews and meta-analyses found that sleep disturbances contributed to higher risk of dementia [3], mental illnesses including anxiety and depression [4], hypertension [5], and cardiovascular disease [6]. Sleep disturbances are also found to have a bidirectional relationship with depression and anxiety [7]. Insomnia and sleep problems were related to high level of anxiety and depression; on the other hand, anxiety and depression were related to insomnia [7].

These health and psychosocial issues are especially pertinent for older people in residential care. The prevalence of sleep disturbance is higher among older people in residential care than among those living in the community [8–11]. A study conducted in Spain showed that a high percentage of nursing home residents (72.1%) were poor sleepers, and poor sleep quality was found to be correlated with decline in functional status [10]. In a European study, the prevalent rate of insomnia in long-term care residents was 24% (ranging from 13% to 30%) [8]. Authors of a narrative review of sleep among long-term care residents in China reported that the prevalence of poor sleep quality ranged from 33% to 73% [9]. In Australia, 47.3% of residents in aged care had poorer night-time sleep quality [12]. The high prevalence of sleep problems in this population is a global health concern that needs to be addressed. Promoting sleep quality may have a positive impact on its associated conditions such as depression, anxiety, and poor QoL.

## Background

Sleep disturbance in the older population is a worldwide health issue that is associated with many health conditions and increased healthcare burdens [1, 13]. Sleep disturbances comprise a broad range of clinical conditions: difficulty in initiating or maintaining sleep, excessive daytime sleepiness, and disrupted sleep–wake patterns [7, 14]. Sleep disturbance in older people is a multifactorial geriatric health issue that calls for a comprehensive treatment approach, with consideration of multiple risk factors such as comorbid diseases, side effects of medications, and psychosocial factors [1, 13]. Integrating conventional medical treatment with complementary therapy to address this clinical issue might be beneficial.

Sleep disturbance in older people is often treated with medications, which have potential adverse effects: drowsiness, poor concentration, loss of memory, and drug dependence [15, 16]. These adverse effects contribute to risks of falls, accidents, and cognitive impairment with long-term use of sleep medications [13, 17]. To avoid the adverse effects of sleep medications, a non-pharmacological treatment approach has been recommended to manage sleep disturbances in older people [17, 18]. Use of complementary and alternative medicine (CAM) may be beneficial for sleep improvement in older people.

Acupressure, application of fingers or thumb pressure on acupuncture points, is a CAM modality which has potential to improve sleep quality [19–21]. Previous research and reviews have shown that acupressure improved sleep quality with no reported adverse effects [19–24]. In addition, acupressure may have potential to reduce the frequency and amount sleep medications used [23, 25]. This can further reduce the cost and side effects of sleep medications. There is also some evidence that acupressure may improve depression [19], anxiety [26] and QoL [21]. However, some studies have methodological limitations as well as variation in acupressure techniques and the selection of acupoints [19, 20, 26]. Future well-designed studies, using standardised treatment protocols, are required to provide evidence of acupressure’s usefulness in clinical practice.

Acupressure is a simple technique which can be administered by nurses with minimal training. Integrating acupressure into aged care settings may be beneficial to promote sleep quality and wellbeing, without having harmful side effects. However, the acceptability and effectiveness of acupressure on sleep improvement remains unclear in the Australia aged care context. The majority of the acupressure studies were carried out in Asian countries (in Taiwan, China, and Hong Kong), and the participants were Chinese who might have had beliefs in traditional Chinese medicine (TCM) [19, 20]. There is a paucity of studies evaluating the outcomes of acupressure in Western countries, including Australia [19, 20]. Acupressure studies conducted in Western populations would enhance the generalisability of the findings. Thus, it is timely to test the efficacy of a standardised acupressure intervention protocol, with well-defined techniques applied on specific acupoints, in residential aged care facilities (RACFs) within the Australia context.

In this study, we focus on sleep quality as the primary outcome because sleep disturbance in this age group is a significant issue which has negative impacts on health status. We will measure anxiety, depression, and QoL as secondary outcomes that are associated with sleep disturbances. The findings of this study will provide important implications and recommendations for future implementation of acupressure intervention in the Australian aged care context, as well as in the worldwide general population with sleep disturbances.

### Acupressure for sleep

Acupressure is the stimulation of acupuncture points (acupoints) on the meridian lines using finger or thumb pressure [27]. The purpose of acupressure is to regulate the vital energy (known as *Qi*) that maintains the person’s health and wellbeing [27]. Manual stimulation of acupoints helps release muscle tension, promotes blood circulation, and strengthens immunity [27, 28]. In the TCM perspective, health is considered as having the normal flow of a vital energy and balance between *Yin* and *Yang* [29]. When *Qi* flows freely, the person is healthy and well-balanced. If there is insufficient *Qi*, or normal movement of *Qi* is impaired, dysfunction occurs, and then physical, emotional, and mental illnesses develop. In the condition of sleep disturbances, it is a result of internal disharmony in *Qi* flow, *Yin* and *Yang* imbalance, and dysfunction of the internal organs [30]. Application of acupressure regulates *Qi* flow within the body or in a specific organ, restoring good health and improving sleep quality [27, 31].

### Acupoints for sleep improvement

In TCM, sleep problems are associated with dysfunction of the heart and other organs, including the spleen, liver, kidneys, and stomach [24, 30]. Therefore, selecting the major acupoint on the heart meridian (HT7) and additional acupoints that are linked to other internal organs (PC6 and SP6) may enhance sleep quality. The acupoints—namely, *Shenmen* (HT7), *Neiguan* (PC6), and *Sanyinjiao* (SP6)—have commonly been used to improve sleep quality [19, 20, 32, 33].

**Fig. 1:**
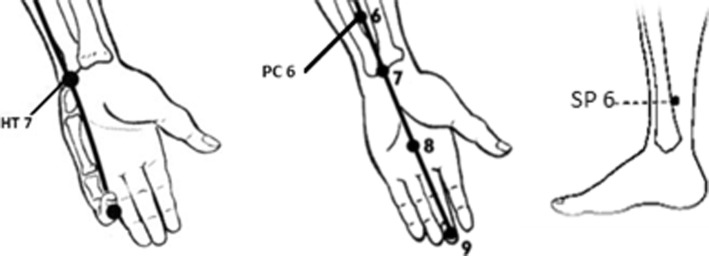
Location of selected acupoints

***Shenmen*(HT7)** is an important acupoint along the heart meridian that passes through and ends in the heart [24]. *Shenmen* is described as the ‘spirit gate’ that is a pathway related to vitality and stabilises mental state, and it is believed to calm the nerves and ease the mind [31]. In TCM, dysfunction of the heart is thought to be a major cause of sleep disturbance. Therefore, pressure applied to the *Shenmen,* the major point of the heart channel, is thought to regulate sleep function [20, 31]. As shown in Fig. 1, HT7 is located on the palmar wrist crease at the depression radial to the proximal border of the pisiform bone [34].

***Neiguan*(PC6)** is the connecting point of the pericardial meridian (PC) that controls the functions of the heart and respiration [35]. This acupoint is commonly used for nausea and vomiting, but it can be used to treat illnesses related to the heart, lungs, and stomach and mental problems. The *Neiguan* regulates the heart *Qi*, opens the chest, harmonises the stomach, and calms the spirit. Acupressure on applied on *Neiguan* could relieve insomnia, dyspnoea, cough, tightness of chest, nausea, vomiting, motion sickness, headache, depression and anxiety [35, 36].

In TCM, dysfunctions of the heart and stomach could be related to sleep disturbances [30]. Thus, the *Neiguan* is a useful point that regulates normal functions of the heart and stomach, thereby relieving sleep problems and associated symptoms. PC6 is located at the anterior aspect of the forearm, between two tendons (the palmaris longus and the flexor carpi radialis), 2-*cun* proximal to the palmar wrist crease (shown in Fig. 1) [34].

***Sanyinjiao*(SP6)** is one of the commonly used acupoints because it is located at the intersection of three *Yin* meridians: the spleen, kidney and liver meridians [35]. This acupoint can be used to treat the conditions related to urogenital disorders, menstruation, childbirth, menopause, indigestion, insomnia and emotional imbalances [35, 36].

According to TCM, sleep disturbance is also caused dysfunctions of spleen, kidney and liver; thus, stimulating the *Sanyinjiao* is commonly applied to improve sleep [24, 30]. SP6 is located on the tibial aspect of the leg, posterior to the medial border of the tibia, 3-*cun* superior to the prominence of the medial malleolus (shown in Fig. 1) [34].

TCM emphasises the individualised nature of therapy, and therefore individualised treatments are given depending on the patient’s conditions [31, 36]. However, in research and clinical practice, standardised acupressure protocols are required, with clearly defined technique and specific acupoints. In this study, finger or thumb pressure applied on three specific acupoints—HT7, PC6 and SP6—is expected to produce a positive effect on sleep quality.

### Study aim and hypotheses

This study aims to evaluate the effects of an acupressure intervention, using three specific acupoints (HT7, PC6 and SP6), on sleep quality, anxiety, depression, and QoL of older people in RACFs in Australia.

### Hypotheses

After the intervention, compared with the control group, the experimental group receiving acupressure would have:

1. Significant improvement of sleep quality,
2. Significant reduction of level of depression and anxiety, and
3. Significant improvement of QoL.

## Methods/design

### Study design

A randomised controlled trial with two parallel groups, pre- and post-test design, is proposed. The study protocol followed the Standard Protocol Items: Recommendations for Interventional Trials (SPIRIT) checklist (Additional File 1). The study participants will be randomly assigned to the intervention group, receiving 12-min acupressure sessions and routine care, or the control group, receiving routine care only.

### Setting and participants

This study will be conducted in three RACFs (RACFs 1, 2 and 3) in New South Wales State, Australia. The selected study venues are facilities accredited by the Aged Care Quality and Safety Commission, Australia. These facilities offer general healthcare for the residents and specialised aged care services, such as dementia care and palliative care. Study participants will be older people residing in the study venues.

### Inclusion and exclusion criteria

The inclusion criteria to participate in this study are (i) aged 65 and above, (ii) a resident of an aged care facility or a retirement village, (iii) have self-reported poor sleep quality, (iv) able to communicate, and (v) able to give written consent to participate and do so. Self-reported poor sleep quality will be identified on the basis of residents’ subjective complaint of disrupted sleep, having sleep problems or poor sleep quality. When potential participants express interest in the study, the researcher will ask them whether they have sleep problems or disturbed sleep.

The exclusion criteria are (i) severe cognitive impairment that would limit the accuracy of assessment; (ii) amputation or recent fracture of upper or lower limbs, because the selected acupoints are located on both hands and legs; (iii) severe illness, presence of blood clotting disorders such as thrombophlebitis, or bleeding disorder such as haemophilia to avoid potential complications; (iv) currently taking blood thinning medication, anti-coagulants and anti-clotting agents; and (vi) presence of skin lesions, infection or inflammation near selected acupoints. Cognitive function level will be determined on the basis of the Mini Mental State Examination (MMSE) record used in the facility. An MMSE score of 9 or lower is considered as severe cognitive impairment in older populations [37].

### Sample size estimation

The sample size estimation is based on a previous study that indicated statistical significance in sleep quality between intervention and control group [23]. The mean difference of sleep quality (Pittsburgh Sleep Quality Index [PSQI] score) between groups was 5, and the pooled standard deviation was 6.35. To detect a mean difference of 5 points (assuming a standard deviation of 6.35), 26 participants in each group are required for a power of 0.80 and *P =* 0.05. Considering a 10% attrition rate, 29 participants in each group, with a total of 58 participants, are required.

### Randomisation

Block randomisation with 1:1 allocation ratio will be used. Block sizes of 4 and 6 will be used. The random sequence numbers will be produced by using an internet tool (www.sealedenvelope.com). Sealed envelopes with sequential numbering will be used to conceal group allocation. Blinding is not possible in this study, because it has limited funding to employ assistants for data collection and acupressure intervention. Therefore, the primary researcher will perform the acupressure and will collect and analyse the data.

### Acupressure intervention and routine care

The participants assigned in the intervention group will receive acupressure and routine care offered in the selected RACFs. The control group will receive routine care only. During the intervention period, participants in both groups will be allowed to take prescribed medications and go for their routine daily activities. The research team does not control any routine activity and prescribed treatment, including sleep medication.

#### Acupressure intervention

The acupressure intervention runs for 4 weeks, with 12-min session to be given three times per week. The acupressure intervention protocol has been developed on the basis of TCM concepts, research evidence [22, 23, 38, 39], and systematic and integrative reviews on acupressure studies [19, 20, 32]. One of the supervisors is a qualified acupressure practitioner. She has supervised the design of the acupressure intervention protocol.

The intervention ‘dosage’ is based on the findings of systematic and integrative reviews [19, 20, 32]. Systematic reviews suggested that acupressure was most effective when applied three or more times per week for a minimum of 3 weeks [20], and a 4-week duration was most commonly used [32]. Finger pressure applied on each acupoint should be a minimum of 1 min [20], and 2–3 min duration applied on each point [19, 32]. In this study, pressure will be applied for 2 min on each acupoint—HT7, PC6 and SP6—on both hands and legs, thus a total of six points for both sides with 12-min duration. Table 1 shows the acupressure intervention protocol.

**Table 1:** Acupressure intervention protocol

Acupressure sessions and length of intervention	• 12-min session, three times per week for 4 weeks • Daytime (9 a.m.–5 p.m.), following the convenient time for study participants
Selection of acupoints	1. Shenmen (HT7) on both hands 2. Neiguan (PC6) on both hands 3. Sanyinjiao (SP6) on both legs
Sequence and time of acupressure application per point	Apply finger pressure for 2 min on each point with the following sequence: 1. PC6 in left hand 2. PC6 in right hand 3. HT7 in left hand 4. HT7 in right hand 5. SP6 on left leg 6. SP6 on right leg
Acupressure technique	• Apply consistent finger or thumb pressure on each point in circular motion • Circular motion in clockwise for 1 min and counter-clockwise for 1 min • Intensity of pressure adjusted according to the person’s level of tolerance

The student researcher (first author) will perform the acupressure intervention. She has experience in general nursing practice and nursing education. She has received training in acupressure technique from a qualified Chinese medicine physician and has performed similar acupressure intervention in her previous postgraduate research. To enhance her skills in acupressure for the present study, she attended acupressure practitioner training in Australia. During the training period, she had practised accuracy of acupoint location and acupressure technique with healthy persons, under supervision of the acupressure trainer, who has extensive experience in acupressure practice and training. Before conducting the acupressure intervention sessions, she rehearsed the acupoint locations and acupressure technique with healthy volunteers, which is verified by the co-researcher, who is expert in acupressure.

To ensure intervention fidelity, the student researcher will perform acupressure following the intervention protocol (Table 1). She will keep field notes that record the date and time of each acupressure session, as well as the observation of participants’ general condition during each acupressure session. To minimise potential discomfort of participants during the treatment, the student researcher will assess the participant’s response, and the intensity of pressure will be adjusted according to the person’s level of tolerance. She will also observe any issue or adverse event that occurs during the intervention period. If any untoward effect occurs, the student researcher will stop doing acupressure on the affected person and report to the nurse in charge and the facility manager. The student researcher and supervisors will have regular meetings to discuss the progress of the study, as well as any issues and concerns related to the study.

#### Routine care

The control group will receive routine care offered in the selected aged care centre (prescribed medications and routine activities). Routine care is the care offered to the participants according to the existing standard practices established in a particular care setting [40]. Routine care is often used for control groups, to compare a specific intervention (e.g., acupressure) with the existing practice in a selected setting [40]. In this study setting, the control group will be given daily routine care, nursing care, general healthcare, and specialised care based on the individual’s needs. The care given to control group will be equal to intervention group, except for the acupressure.

### Recruitment

The recruitment procedure will be carried out by the student researcher. The recruitment poster will be posted in the facility to advertise the research project, and flyers will be placed in reception and common areas of the facility. The student researcher will also promote the study during their routine social activities. She will present the study and then ask the potential participants for their expression of interest, either verbally or by contacting her via telephone or email. When the residents contact the student researcher and express interest in participating, the student researcher will explain the details of study procedures and provide them the participant information sheet and consent form. The researcher will assure that their participation in the study or decision to decline to participate will not affect the services they receive in the facility. If they agree to participate, information about inclusion and exclusion criteria will be identified on the basis of their medical records. Residents who give consent and meet the inclusion criteria will be enrolled in the study, given with a specific identification number (e.g., 001, 002). They will then be assigned to the intervention or control group, using random sequence numbers generated by the internet tool.

### Data collection

Data collection will be carried out by the researcher for pre-test, post-test, and 2-week post-test. The data collection timeline is shown in Table 2. The baseline data collection (pre-test, T0) will be conducted before commencing the acupressure intervention, using a structured questionnaire for demographic and clinical data and validated assessment tools for sleep quality, depression, anxiety and QoL. Demographic and clinical data include age, sex, marital status, number of children, education level, residential status, length of stay in facility, source of funding, presence of chronic disease, and medications.

**Table 2:** Data collection timeline

Stages	Enrolment	Pre-test T0	4-Week acupressure intervention				Post-test, T1		Follow-up, T2
Time point	Week 0	Week 1	Week 2	Week 3	Week 4	Week 5	Week 6	Week 7	Week 8
Recruitment and consent	4–6 weeks								
Baseline assessment: - Demographic and clinical data - ActiGraph - PSQI, HADS, OPQOL-Brief		X							
Intervention: IG: acupressure + routine care CG: routine care			X	X	X	X			
Post-test outcome assessment: - ActiGraph - PSQI, HADS, OPQOL-Brief							X		
Follow-up assessment: - ActiGraph - PSQI, HADS, OPQOL-Brief									X

*CG* Control group, *HADS* Hospital Anxiety and Depression Scale, *IG* Intervention group, *OPQOL-Brief* Older People’s Quality of Life questionnaire–brief version, *PSQI* Pittsburgh Sleep Quality Index

The outcome assessments will be conducted at the end of intervention (post-test, T1) and 2 weeks post-intervention (post-test, T2). The participants will receive only routine care after the intervention period. The purpose of the follow-up test (T2) is to assess whether the treatment effect of acupressure can be retained 2 weeks after the intervention. For the control group, baseline and outcome data (T0, T1 and T2) will be collected using the same set of questionnaires and following the same time frame as in the intervention group. The student researcher will provide the assessment tools to the participants and assist them in completing the questionnaires if needed.

### Outcome assessments

The primary outcome for the acupressure intervention is sleep quality, which will be measured using the PSQI [41] and an objective sleep measurement device (ActiGraph, Pensacola, FL, USA). The secondary outcome measures include anxiety, depression and QoL. The Hospital Anxiety and Depression Scale (HADS) [42] will be used to measure anxiety and depression, and the brief Older People’s Quality of Life Questionnaire (OPQOL-Brief) [43] will be used to measure QoL.

#### Pittsburgh Sleep Quality Index

The PSQI measures a person’s subjective sleep quality over the previous 1-month time period. It consists of 19 items with seven sub-scores: subjective sleep quality, sleep latency, sleep duration, sleep efficiency, daytime dysfunction, sleep disturbances and use of sleeping medications [41]. The total score ranges from 0 to 21; higher scores indicates worse sleep quality, and a score greater than 5 indicates poor sleep quality [41].

The original version of the PSQI is a valid and reliable tool that is widely used to measure subjective sleep quality, with Cronbach’s α of 0.83 in healthy adults and individuals with sleep complaints and psychiatric symptoms [41]. The construct validity and internal consistency reliability in older adults was shown to be adequate, with the Cronbach’s α = 0.69 in older men [44] and α = 0.72 in older women [45].

In this study, sleep quality will be assessed before, immediately after and again 2 weeks after the intervention. To be consistent with the period between ratings, the time frame of the PSQI is modified as ‘during past two weeks’ instead of ‘during past month’. The modified version of the PSQI assesses sleep quality within the past 2 weeks.

#### Objective sleep measurement (ActiGraph)

The actigraphy device (ActiGraph GT9X Link) is equipped with a sensor that measures objective sleep patterns of a person. The ActiGraph is found to be a valid tool to determine the changes in sleep quality induced by specific intervention; therefore, it is suggested as a useful device for intervention studies [46]. To obtain reliable sleep data, the participants need to wear the device for a 1-week period [46, 47]. Therefore, the ActiGraph data will be recorded for 7 days before the intervention (T0), after the intervention (T1), and for 2 weeks post-intervention (T3). Participants will be asked to wear the device on the dominant wrist for 7 days and nights continuously, with it to be taken off only when showering. The data recorded by the ActiGraph will be downloaded and analysed using the ActiGraph software (ActiLife 6).

#### Hospital Anxiety and Depression Scale

The HADS is a tool for detecting emotional states of depression and anxiety in an adult population, and it is recommended for use in examining changes over time through intervention trial [42, 48]. It includes 14 items: seven items for anxiety and seven for depression. The HADS uses a 4-point Likert scale (0–3) for each item. The total score of anxiety and depression subscales ranges from 0 to 21; a higher score indicates higher level of depression or anxiety [42]. The HADS has shown good psychometric properties with good internal consistency in older adult samples: α = 0.84 for the anxiety subscale, and α = 0.75 for the depression subscale [48].

#### Brief Older People’s Quality of Life Questionnaire

The OPQOL-brief is a short version of the 35-item OPQOL questionnaire [43]. The total score ranges from 13 to 65; a higher score represents higher QoL. The OPQOL-brief was shown to be a reliable tool (α = 0.85), and it is a suitable tool for outcome assessment of social and health interventions in older people [43].

### Data analysis plan

Data will be analysed using IBM SPSS Statistics version 24.0 software (IBM, Armonk, NY, USA). The researcher will perform data entry using participants’ code numbers. The data will be explored and assessed for missing values, outliers, extreme values and normal distribution. Descriptive statistics will be used to explore baseline data. The generalised estimating equation (GEE) for repeated measurements will be used to examine the intervention effects on sleep quality, depression, anxiety and QoL. The primary outcome will be the treatment effect of acupressure on the post-treatment (T1) sleep quality scores measured by the PSQI and the ActiGraph. The post-treatment scores for the HADS and QoL will be used as secondary outcomes. The outcome data measured at 2-week follow-up will be analysed to determine the treatment effect of acupressure on the outcome variables (PSQI, ActiGraph, HADS and QoL scores) 2 weeks after the intervention.

**Fig. 2:**
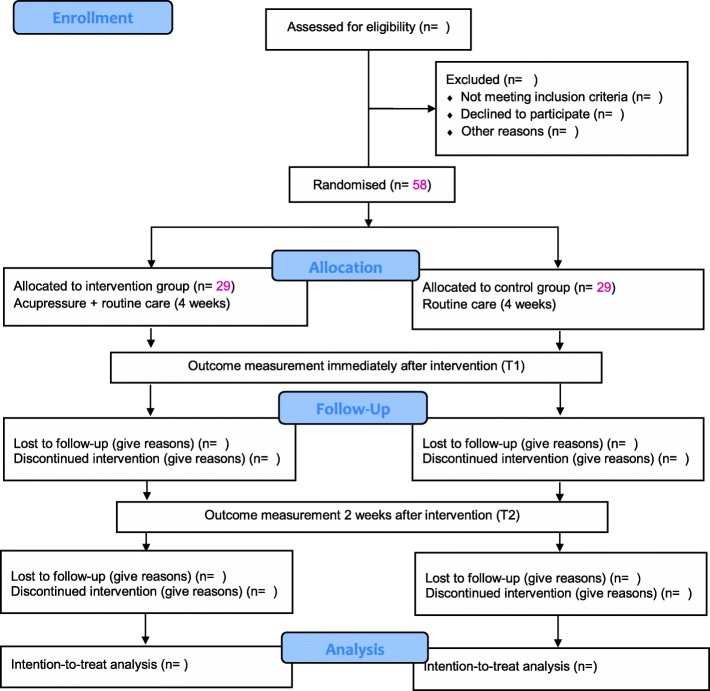
Study flow diagram

Adjusted analyses will be conducted for age group; sex; use of sleep medication; and baseline PSQI, HADS and QoL scores. Sub-group analyses will be conducted to determine the differences in treatment effect of acupressure based on age group (65–85, 85–100, > 100 years) and use of sleep medication (yes/no). The correlation between outcome variables will be analysed (baseline, post-treatment, and 2-week follow-up), using Pearson or Spearman rank-correlation, depending on whether the data meet statistical assumptions. The statistical significance will be *P* < 0.05. The intention-to-treat method will be used that analyses participants according to their treatment allocation, regardless of deviation from the intervention protocol [49]. Missing-at-random assumption will be used to handle missing data in GEE analysis. The study flow diagram is shown in Fig. 2.

### Ethical consideration

This study will be conducted with approval from the University Human Research Ethics Committee, granted on February 2019 (approval reference no. H-2018-0345). Permission and organisational consent will be obtained from the director or manager of the facilities. This study will be conducted following the ethical standards established by the National Health and Medical Research Council, Australia [50]. The researchers will ensure that participation in this study is on a voluntary basis, and the participants can withdraw from the study at any time before completion of the study without any impact on the care they receive. Residents who agree to participate will be required to sign a written consent form before enrolling in the study. Participants’ confidentiality will be ensured by using identification code numbers. Participants’ consent forms and completed demographic data and assessment tools will be stored in a locked office cabinet that can be accessed only by the student researcher and supervisors involved in the study. All electronic information and files containing electronic data will be saved on a password-secured computer.

### Trial registration

This study is prospectively registered in the Australian New Zealand Clinical Trials Registry (ACTRN12619000262101; registered on 20 February 2019). At the completion of the study, a summary of the results and changes from initial protocol will be updated in the trial registry/ (http://www.anzctr.org.au/ACTRN12619000262101.aspx).

## Discussion

This study is designed to investigate the effects of an acupressure intervention on sleep quality and associated conditions, namely depression, anxiety, and QoL, among older people living in RACFs in an Australian context. To the researchers’ knowledge, this is the first study evaluating the outcomes of an acupressure intervention in an Australian aged care context. This study uses a well-defined acupressure intervention protocol, focusing on three specific acupoints—HT7, PC6 and SP6—for promoting sleep quality. In addition, this study uses both subjective and objective sleep measurements (questionnaire and ActiGraph), which can provide a valid outcome of sleep quality. The secondary outcomes for depression, anxiety, and QoL are measured using the valid and reliable assessment tools. The findings from this study will provide good evidence of the usefulness and effectiveness of acupressure regarding the sleep quality and wellbeing of older people in the Australian context.

With regard to participant characteristics, Australian older people living in aged care facilities or retirement villages are aged 65 and above and have diverse socio-demographic backgrounds. They are living with chronic conditions and taking multiple prescribed medications. The characteristics of the study sample are similar to the wider population in RACFs. The study venues are also representative of the Australian aged care context. To ensure recruiting of representative samples, the student researcher will promote the project and provide study information to potential participants while the residents are attending their routine activities in the study venues (e.g., morning exercise, afternoon tea, craft sessions). She will also connect with facility staff and the care team to get support for recruiting participants.

When analysing the outcome data, robust statistical methods will be used, including adjusted and sub-group analyses. There may be correlation between outcome variables, such as improvement of sleep quality may be correlated with improvement in depression, anxiety, and QoL. Therefore, we will conduct correlation analysis of outcome measures, and the results will be discussed further in reporting the findings in a paper. This study will not include a cost-utility analysis. If the older people have better sleep, it could lead to better wellbeing and less dependence on medication, and it may reduce some healthcare costs.

Stimulating acupoints using finger or thumb pressure is a simple technique that is easy to learn. Nurses, caregivers and even family members can learn basic acupressure techniques and apply acupressure for older people. Therefore, integration of acupressure in aged care and clinical practice is feasible and could be beneficial for promoting sleep and wellbeing of older people and the general population.

### Limitations of the study

The researchers and participants are not blinded to the study. The student researcher will carry out data collection, acupressure intervention, and data analysis with guidance from her doctoral degree supervisors. The researcher will comply with the codes of conduct and research integrity to minimise study bias. She will administer the questionnaires without influencing participants’ responses to the questions. The identification code numbers will be used throughout collecting and analysing data.

Sham intervention and attention control are not used for the comparison group. Because only one researcher will perform acupressure, it is not possible for her to perform sham acupressure or attention control activities concurrently with the acupressure group. This is a study for doctoral program project that evaluates the potential efficacy of acupressure in promoting sleep quality and associated outcomes. If the intervention is found to be effective, further funding will be sought for a large-scale study, with sham intervention and blinding procedures.

### Trial status

Participant recruitment commenced in March 2019 (protocol version 3, dated 26 November 2018). Recruitment has been slower than expected. Many residents had little knowledge of acupressure, and they had low interest. Some of them were unable to commit their time to the intervention, and some interested participants did not meet eligibility criteria. We extended the recruitment sites in RACF 2 (protocol version 4, dated 29 May 2019) and RACF 3 (protocol version 5, dated 23 July 2019). The project has been completed in 15 April 2020. The study findings will be published in journals and presented at local and international conferences.

## Conclusion

This study targets a poorly met healthcare need of older people who are experiencing sleep disturbance and its negative consequences. Acupressure is a simple technique that can be easily applicable in practice settings to promote the sleep and wellbeing of older people. If the results show the usefulness of acupressure to promote sleep and wellbeing, it will provide broad implications for the use of acupressure in aged care and other clinical practices. The standardised acupressure protocol and specific acupoints used in this study may contribute to the further development of acupressure intervention for promoting sleep and wellbeing. Therefore, this study’s findings will be applicable to the global ageing population as well as those in the general population who are experiencing sleep disturbances.

## Supplementary information

**Additional file 1.** SPIRIT checklist.

## Data Availability

The study findings will be published in journals and presented at local and international conferences. The datasets analysed during this study will be available from the corresponding author upon reasonable request.

## Abbreviations

CAM: Complementary and alternative medicine
GEE: Generalised estimating equations
HADS: Hospital Anxiety and Depression Scale
HT: Heart meridian
MMSE: Mini Mental State Examination
OPQOL: Older People’s Quality of Life Questionnaire
PC: Pericardial meridian
PSQI: Pittsburgh Sleep Quality Index
QoL: Quality of life
RACF: Residential aged care facility
SP: Spleen meridian
SPIRIT: Standard Protocol Items: Recommendations for Interventional Trials
TCM: Traditional Chinese medicine
